# The pathology trope: How television undermines the diagnostic infrastructure of modern medicine

**DOI:** 10.1016/j.acpath.2026.100297

**Published:** 2026-09-11

**Authors:** Adil Menon

**Affiliations:** a Department of Pathology, Case Western Reserve University, Cleveland, OH, USA; b Department of Pathology, MetroHealth Medical Center, Cleveland, OH, USA

**Keywords:** Hidden curriculum, Medical education, Medical television, Pathology, Precision oncology, Recruitment, Stereotypes, Workforce

## Abstract

Television medical dramas have long perpetuated a cultural hierarchy among medical specialties, and pathology has been a consistent target. From overtly comedic portrayals of incompetent physicians reassigned to the morgue to subtler character constructions that equate interest in pathology with professional disengagement, these narratives encode and broadcast the assumption that pathology is a lesser specialty — a destination for those who lack the commitment or endurance demanded by “real” clinical medicine. This piece examines the most recent iteration of this trope in the television series *The Pitt,* in which a medical student's stated interest in pathology is narratively tethered to her willingness to leave the emergency department on time, and contextualizes it within a broader pattern spanning *Scrubs, The Good Doctor, Grey's Anatomy, House,* and the forensic pathology subgenre. Drawing on workforce data, recruitment literature, and the documented scope of pathology and laboratory medicine — which underpins an estimated 60–70% of all clinical decisions — this article argues that the pathology trope is not merely a harmless fictional convention but an active contributor to the specialty's recruitment crisis, public invisibility, and cultural devaluation. At a time when the United States faces a projected deficit of more than 5000 pathologists by 2030, the cultural signals embedded in prestige television carry measurable consequences for the diagnostic infrastructure of modern medicine.

*The New York Times* recently produced an article focused on the controversy surrounding Joy Kwon's departure scene in *The Pitt.* When invited to be part of the conversation, I shared part of this perspective — but the format and scope of the interview did not allow me to fully articulate the deeper cultural machinery at work. This piece provides the fuller articulation.

In the final moments of a chaotic shift, the camera lingers on Joy Kwon as she gathers her things and walks out of the emergency department — on time. Around her, colleagues stay. They bleed into the next hour, the next crisis, the next patient. The audience is meant to notice. And in noticing, to judge.

This is not a subtle narrative choice. Joy Kwon has explicitly expressed interest in pathology. And so her departure — framed against the visible sacrifice of her colleagues — is not merely an individual-character beat. It becomes a statement about the specialty itself. The audience is invited to draw a line: she is the one who wants pathology, and she is the one who leaves. The implication is seamless and corrosive. Her behavior is made legible through her specialty interest, and her specialty interest is made legible through her behavior. Each confirms the other. The result is that pathology is painted as the destination for physicians who do not stay, do not sacrifice, and do not care enough.

This is not new. It is the latest iteration of one of medical television's most durable and damaging tropes: the idea that real doctors stay, and lesser ones leave — and that the lesser ones end up in pathology. The trope often takes the form of a repeated narrative formula: a character who fails or is deemed unfit for a “primary” clinical specialty is redirected into pathology as a consolation or last resort. In *Scrubs*, Doug Murphy is a running gag — a physician so catastrophically incompetent at patient care that patients literally die under his watch, and his eventual reassignment to pathology is played as both a punchline and a mercy, the show's way of saying that the morgue is where you end up when you cannot be trusted with the living. In *The Good Doctor*, the trope operates on multiple registers. Shaun Murphy, a surgical resident with savant-level diagnostic brilliance, is temporarily reassigned to pathology as a form of professional exile — a demotion from the “real” work of surgery, a place to be sidelined rather than celebrated. But the show's treatment of pathology extends beyond career narrative into its interpersonal dynamics. Shaun's romantic partner, Carly Lever, is a pathologist — and the relationship is structured in ways that consistently subordinate her professional identity to his. Her expertise is instrumentalized when it serves his surgical cases but otherwise rendered peripheral. The relationship ultimately fails, and the narrative arc implicitly frames the incompatibility not as a mismatch of personalities but as a mismatch of professional gravitas — the surgeon-savant and the pathologist occupy different tiers of the show's moral and intellectual universe, and the audience is invited to feel that the relationship was always asymmetric. The message is not spoken, but it is legible: the pathologist is not quite an equal. In both of these narrative functions — exile and romantic subordination — pathology is not portrayed as a destination chosen by capable physicians drawn to diagnostic complexity. It is portrayed as a lesser orbit. *Grey's Anatomy* built an entire mythology around surgery as the pinnacle of medical achievement, rendering nonprocedural specialties invisible or irrelevant. *House*, for all its diagnostic brilliance, drew its intellectual prestige from the bedside while the laboratory professionals who would actually generate the answers remained faceless.

But television's distortion of pathology is not limited to the clinical trope. An equally extensive body of work features forensic pathologists — from *Quincy, M.E. to CSI to Bones* — complete with its own collection of unrealistic portrayals concerning professional behavior, scope of practice, impossibly advanced technology, and turnaround times that bear no resemblance to actual laboratory operations. While these shows occasionally lend forensic pathology a certain dramatic prestige, they ultimately compound the problem: by overemphasizing forensics, they obscure the true breadth of the field — the surgical pathology, the molecular diagnostics, the transfusion medicine, and the clinical laboratory science — and reduce the public's understanding of pathology to autopsies and crime scenes. The forensic trope and the clinical trope are two sides of the same distortion. One diminishes pathology by making it a punchline; the other distorts it by making it unrecognizable.

The *Pitt* is subtler than its predecessors, and that is precisely what makes it more effective. Joy Kwon is not mocked. She is not told to “go into pathology” as an insult. She already wants to. And so the show does not need to name the hierarchy — it activates it through character design. To be fair, some commentators have noted that Kwon is portrayed as brilliant and have read her departure as a heroic — a principled stand against a toxic work environment rather than a mark of disengagement. That reading is not unreasonable. But it does not neutralize the trope; it refines it. The structural message of the scene is not that Joy Kwon is stupid or lazy. It is that the future pathologist is the one who hits her limit first — the one whose threshold for the demands of acute clinical medicine is lower than that of her peers who stay. The character may be sympathetic, even admirable in isolation, but the narrative still encodes a hierarchy of endurance, and it is the pathology-bound student who falls short of it. A medical student interested in pathology is made the vehicle for every assumption the audience already holds — that pathology attracts the disengaged, the clock-watchers, the ones who would rather leave than lean in. By tethering her specialty interest to her departure, the show encodes the stereotype into her identity. Millions of viewers — many of whom will never meet a pathologist, never learn what pathologists do, and never understand how often their or a loved one's diagnosis depended on one — absorb this association without resistance. This is the architecture of the pathology trope, and *The Pitt* builds it perhaps more efficiently than any show before it.

In reality, pathology and laboratory medicine underpin the majority of all clinical decisions. A *Lancet* commission described pathology and laboratory medicine as “a highly complex and integrated set of medical and technical subdisciplines” that are “crucial” to the delivery of effective healthcare, estimating that approximately 60–70% of clinical decisions rely on laboratory results.^1^ Virtually every cancer diagnosis, for example, depends on pathological interpretation. Molecular pathology now drives precision oncology — determining which patients receive targeted therapies, which tumors are reclassified based on genomic profiling, and which treatment strategies are selected at multidisciplinary tumor boards.^2^, ^3^, ^4^, ^5^ The European Society of Pathology has formally affirmed that interpretation of somatic molecular alterations in cancer belongs to the scientific domain of pathology, and that pathologists must lead this work to ensure quality and clinical accuracy.^6^

These are not basement specialties. They are the diagnostic infrastructure on which the rest of medicine depends.

But there is another dimension to the Joy Kwon scene that deserves attention: the implicit suggestion that a physician interested in pathology must not have worked as hard to get there — that her desired seat in pathology will be markedly easier to earn. This is flatly wrong.

Every physician in that room — regardless of what specialty they will ultimately practice — endures the same gauntlet to arrive there. Four years of medical school. The same foundational curriculum. The same anatomy labs, the same clinical clerkships, the same overnight call as third-year students on surgery and internal medicine, the same shelf exams, the same step examinations. A future pathologist rotating through the emergency department as a medical student is held to the same standard as a future surgeon or internist. The evaluations received on every rotation — not just the ones aligned with an eventual career — carry weight. A national survey of residency program directors across 21 specialties found that the top selection criteria were grades in required clerkships, United States Medical Licensing Examination (USMLE) step scores, grades in senior electives, and number of honors grades.^7^ These criteria applied across specialties regardless of competitiveness.^7^ With the transition of USMLE step 1 to pass/fail scoring, clerkship performance has become even more consequential — program directors across all specialties now place greater emphasis on core clerkship grades, narrative assessments, and subinternship evaluations.^8^^,^^9^ Every rotation matters. Every evaluation counts. Joy Kwon cannot coast through her emergency medicine rotation any more than her surgery-bound colleague can coast through psychiatry. Her interest in pathology does not exempt her from the full rigor of clinical training.

And the notion that pathology is a fallback — a refuge for those who “couldn't cut it” — is increasingly divorced from reality. The pathology workforce in the United States has been in sustained decline, with the number of active pathologists falling from 15,568 to 12,839 between 2007 and 2017 — a 17.5% decrease — even as the diagnostic workload per pathologist rose by nearly 42%.^10^ The number of new board-certified pathologists entering the workforce is approximately 600 per year, but the field experiences a net loss of roughly 270 pathologists annually beyond that number, meaning residency positions would need to increase by 45% simply to offset current attrition.^10^ A 2021 College of American Pathologists survey found that 55.7% of pathology practices sought to hire at least one pathologist that year, up from 45.3% in 2017, with practice leaders anticipating continued strong demand — and concern that demand may outstrip supply.^11^ Predictive models have forecast a deficit exceeding 5000 full-time equivalent pathologists by 2030.^12^

This is not a specialty in retreat. It is a specialty in crisis-level demand — one that increasingly requires not only diagnostic expertise but computational fluency, genomic literacy, and the ability to function as a high-level consultant across oncology, infectious disease, and transfusion medicine. The training itself reflects this complexity: pathology residency requires 16,000 hours of training to achieve board eligibility, with competency milestones spanning patient care, medical knowledge, systems-based practice, and interpersonal communication — the same Accreditation Council for Graduate Medical Education (ACGME) competency domains applied to every other specialty.^13^^,^^14^ More than a third of pathology residents report experiencing burnout during training, with workload identified as the leading contributing factor — mirroring the pressures seen across all of graduate medical education.^15^^,^^16^

Yet the cultural hierarchy persists, and television both reflects and reinforces it. When *The Pitt* frames leaving on time as a character flaw — and assigns that flaw specifically to the character who wants pathology — it does more than judge one fictional physician. It tells the audience that the specialty itself produces this kind of doctor.

What makes Joy Kwon's storyline particularly insidious is its emotional sophistication. Older versions of this trope were crude: Doug Murphy's body count played for laughs, Shaun Murphy's exile played for pathos, Carly Lever's professional identity dissolved into a romantic subplot, the “couldn't cut it” dismissal, the autopsy-as-outsider visual shorthand. *The Pitt* operates through character construction. It does not argue that pathology is lesser. It simply builds a character whose stated interest in pathology and whose willingness to leave on time become indistinguishable — two facets of the same implied deficiency. This is more effective propaganda precisely because it feels like character development rather than ideology. The audience does not feel manipulated. They feel like they are observing a truth about who Joy Kwon is. And by extension, about what pathology is.

And this is where the cultural cost becomes measurable. The pathology recruitment literature reveals a field already fighting an uphill battle against invisibility and stereotype — and television is part of the machinery that sustains both.

A focus group study of senior medical students found that the single most powerful anti-pathology influence on residency choice was that pathology was “utterly invisible in clinical practice” — most students did not consider and then reject pathology; they ignored it entirely.^17^ When students did form impressions, those impressions were shaped by negative stereotypes: perceptions of pathologists as introverted, disengaged, and “in some ways nonmedical.” An essay-based survey of first-year clinical residents found that the dominant barriers to choosing pathology were perceptions that it “lacks practical application to patient care” or provides “no real help” — beliefs the authors noted were “based more on stereotypes than reality."^18^ A study of Canadian postgraduate trainees found that clinical residents who rejected pathology cited “misconceptions and stereotypes about pathology, including misunderstandings about the role of pathologists and the nature of pathology practice."^19^ Within the US match data between 2010 and 2019, 40.5% fewer senior US allopathic medical students pursued pathology in the Main Residency Match.^20^

Television medical dramas do not merely reflect this hierarchy — they broadcast it to millions of viewers who will never encounter the hidden curriculum of a medical school but who will absorb its conclusions. When *The Pitt* makes Joy Kwon's interest in pathology legible only through her willingness to leave, it does not just reinforce a stereotype within medicine. It exports that stereotype to the culture at large, where it calcifies into common knowledge: pathology is where the uncommitted go. And for a specialty already struggling against invisibility — where 87.6% of medical students in one study had never spent significant time in a pathology laboratory, where the public cannot correctly identify who makes their cancer diagnosis, where the workforce is projected to face a deficit of over 5000 pathologists by 2030 — every cultural signal matters.^12^^,^^21^ Every fictional physician who drifts into pathology as a consolation prize makes the next real physician less likely to choose it as a calling.

*The Pitt* did not invent this problem. But it refined the delivery mechanism. And in doing so, it contributed — with all the reach and emotional authority that prestige television commands — to a cultural narrative that is actively undermining the diagnostic infrastructure of modern medicine.

## Declaration of competing interest

The author declares no competing interest. The views expressed in this article are solely those of the author and do not represent the official position of any institution or organization.
